# Core self-evaluation and learning burnout among first-year medical students: a three-wave longitudinal study on the mediating roles of anxiety-related distress and problematic digital media use

**DOI:** 10.3389/fmed.2026.1818069

**Published:** 2026-05-21

**Authors:** Shoukai Yang, Xudong Yang, Xiang Li, Li Chen, Yawen Zheng, Zheru Dai

**Affiliations:** 1 Department of Psychiatry, The Third People’s Hospital of Huzhou, The Affiliated Hospital of Huzhou University, Huzhou, China; 2 School of Educational Science, Hunan Normal University, Changsha, China; 3 College of Education, Wenzhou University, Wenzhou, China; 4 School of Mental Health, Wenzhou Medical University, Wenzhou, China; 5 Lishui Second People’s Hospital, Wenzhou Medical University, Lishui, China; 6 Department of Student Affairs, Wenzhou Medical University, Wenzhou, China

**Keywords:** anxiety-related distress, core self-evaluation, first-year medical students, learning burnout, problematic digital media use

## Abstract

**Background:**

First-year medical students are at high risk of learning burnout due to the substantial academic and psychological demands of medical education. This study examined whether core self-evaluation (CSE) was prospectively associated with later learning burnout, and whether anxiety-related distress and problematic digital media use served as parallel mediating pathways.

**Methods:**

A three-wave longitudinal survey was conducted among 4,890 first-year medical students at 6-months intervals. CSE and baseline learning burnout were assessed at Time 1 (T1), anxiety-related distress and problematic digital media use at Time 2 (T2), and learning burnout at Time 3 (T3). Structural equation modeling was used to test a baseline-adjusted parallel mediation model.

**Results:**

After controlling for baseline learning burnout, the direct path from T1 CSE to T3 learning burnout was no longer significant. However, T1 CSE was indirectly associated with T3 learning burnout through both T2 anxiety-related distress and T2 problematic digital media use. Lower CSE was associated with higher anxiety-related distress and greater problematic digital media use, which were in turn associated with higher levels of later learning burnout.

**Conclusion:**

After accounting for baseline burnout, the association between CSE and later learning burnout was primarily reflected through emotional and behavioral pathways. Anxiety-related distress and problematic digital media use may therefore represent meaningful targets for the early identification and prevention of learning burnout in first-year medical students.

## 1 Introduction

In health science education, the well-being and health of students are not only crucial for individual development but also serve as the foundation for ensuring the quality of future healthcare services and the overall sustainability of the healthcare system. However, the World Health Organization has explicitly identified occupational health risks within academic environments as a significant global challenge (1). Among these risks, learning burnout, characterized by a negative attitude and exhausted behavior due to excessive academic pressure or lack of interest (2), has become a pervasive issue in higher education, particularly within the medical field (3).

Medical training is typically characterized by long training periods, heavy academic workloads, rapidly evolving knowledge, and high-stakes assessment demands (4, 5), placing students under sustained pressure and increasing their risk of emotional exhaustion, cynicism, and reduced academic efficacy (4). For first-year medical students, this risk may be further intensified by the rapid transition from general secondary education to professional medical training (6). In the Chinese educational context, where examination performance and academic competition are strongly emphasized (7), the prevalence of learning burnout among medical students has reached an alarming range of 25.8%–52.1% (8). Given that learning burnout is closely associated with academic impairment, greater psychological distress, and even increased suicide risk (9–11), identifying its prospective risk factors and pathways among first-year medical students is of clear theoretical and practical importance.

Core self-evaluation (CSE) is a higher-order self-evaluative personality trait that reflects individuals’ fundamental appraisals of their own worth, competence, and capabilities (12). Previous research has shown that higher CSE is generally associated with more adaptive learning attitudes, better emotional regulation, and stronger academic adjustment (5, 13). According to Self-Determination Theory (SDT), CSE serves as a critical psychological resource for satisfying the need for competence (14). In the high-pressure medical school environment, students with high CSE tend to experience greater autonomy, competence, and relatedness in their learning, which enhances intrinsic motivation and emotional regulation, thereby reducing learning burnout (15). Conversely, students with low CSE may experience less autonomy, competence, and relatedness, increasing the risk of learning burnout (16). Longitudinal studies have indicated that self-esteem (one of the core components of CSE) significantly predicts subsequent academic self-efficacy among college students (17), suggesting that CSE could serve as a predictor of learning burnout. Therefore, we propose the hypothesis 1: CSE will significantly and negatively predict subsequent learning burnout among first-year medical students.

From the perspective of stress and coping theory (18), low CSE may leave individuals more likely to appraise academic demands as threats rather than manageable challenges, thereby increasing their vulnerability to persistent psychological strain and anxiety-related distress. This vulnerability may be particularly salient among first-year medical students, who must simultaneously adapt to heavier coursework, changing academic roles, and new social environments (4). In the present study, anxiety-related distress was conceptualized as a broader anxiety-related domain rather than a narrow or unitary clinical anxiety construct (19–21). Specifically, state anxiety reflects general affective-physiological arousal, social anxiety reflects interpersonal-evaluative concerns and related avoidance tendencies, and fear of missing out (FoMO) reflects a social-cognitive anxiety-related concern about missing rewarding or socially meaningful experiences. Although these three indicators are conceptually distinct, they may co-occur during periods of heightened adjustment stress and jointly reflect a broader latent distress response. Prior research has shown that anxiety-related experiences and psychological distress are closely associated with learning burnout (22, 23). Moreover, studies on medical students have revealed that psychological distress mediates the connection between personality traits–such as neuroticism, a facet of CSE–and learning burnout (6). Building on existing literature, it is reasonable to propose Hypothesis 2: anxiety-related distress may mediate the relationship between CSE and subsequent learning burnout in first-year medical students.

In addition to emotional distress, lower CSE may also be linked to later learning burnout through maladaptive behavioral responses. From the perspective of SDT (14), students with lower CSE may be more likely to experience reduced feelings of competence and self-worth, and may therefore turn to external compensatory behaviors to relieve stress and regulate negative emotions. In the highly digitalized context of university life, short-video use and online gaming, with their immediate rewards, immersive experiences, and low barriers to engagement, may serve as particularly accessible forms of such compensatory coping (24, 25). When digital media engagement becomes characterized by impaired control, escapist motivation, and excessive immersion, it may develop into problematic digital media use (24). Problematic digital media use may in turn increase the risk of learning burnout by consuming study time and self-regulatory resources, disrupting sleep, and undermining sustained attention and academic engagement. For instance, a study involving 2,260 Chinese adolescents indicated that problematic internet use could serve as a predictor of future learning burnout (26). In the present study, we focused on problematic short-video use and problematic online gaming as two representative forms of digital behavior. Although they differ in specific content and mode of interaction, both reflect shared features of problematic digital media engagement, including impaired control, escapist use, and risk of functional impairment (27–29). Moreover, previous empirical research indicated that low CSE associated with problematic internet use (30, 31). Therefore, we propose Hypothesis 3: problematic digital media use would act as a mediator in the relationship between CSE and subsequent learning burnout.

Taken together, students with lower CSE may be more likely not only to experience anxiety-related distress but also to engage in maladaptive behavioral coping, such as problematic digital media use. These two pathways may reflect emotional and behavioral responses to demanding academic environments, and both may be associated with later learning burnout. It should be noted, however, that although anxiety-related distress and problematic digital media use may be theoretically related, the two mediators were assessed at the same time point in the present study. Therefore, rather than treating them as an empirically established sequential process, the present study more cautiously conceptualized them as two related but relatively distinct parallel indirect pathways. From this perspective, anxiety-related distress and problematic digital media use may represent emotional and behavioral pathways, respectively, linking lower CSE to later learning burnout.

**FIGURE 1:**
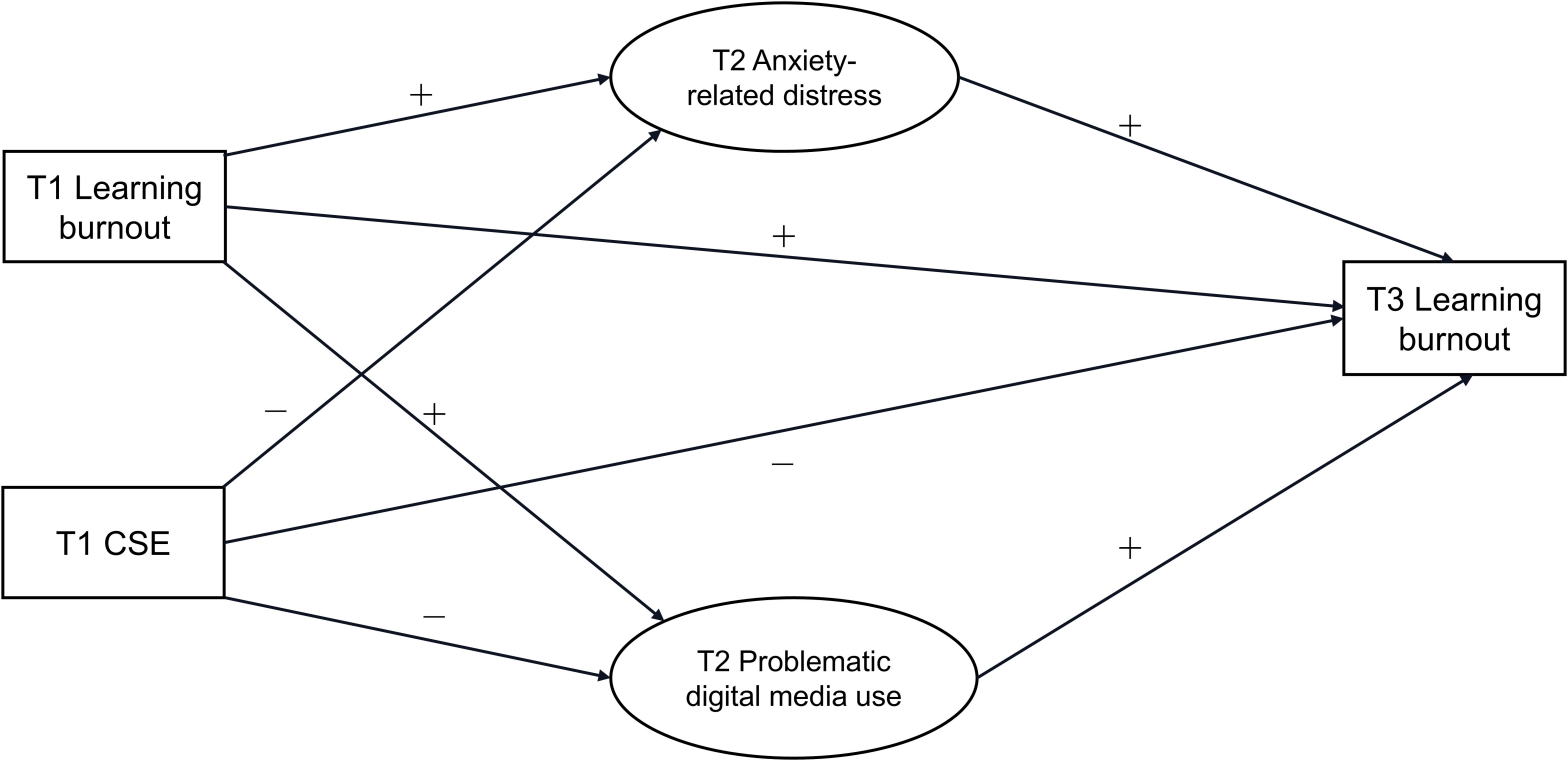
Conceptual framework of the baseline-adjusted parallel mediation model.

Although research on learning burnout among medical students has continued to grow, the existing literature still relies largely on cross-sectional designs, limiting stronger inferences about prospective associations among the variables. In addition, relatively few studies have simultaneously examined emotional and behavioral adjustment pathways within the same framework, and even fewer have considered baseline learning burnout when explaining later burnout. These issues are particularly important for first-year medical students, who are undergoing both academic and social transitions. To address these gaps, the present study used a three-wave longitudinal design with a sample of 4,890 first-year medical students to examine whether T1 CSE was prospectively associated with T3 learning burnout after accounting for baseline learning burnout, and whether anxiety-related distress and problematic digital media use functioned as two parallel indirect pathways in this association. In doing so, this study aimed to provide more rigorous longitudinal evidence on early psychological risk factors and potential pathways underlying learning burnout among first-year medical students (see Figure 1).

## 2 Materials and methods

### 2.1 Participants and procedure

Participants were recruited from four medical universities in the province of Zhejiang, China, and participated in a three-wave longitudinal online survey. Data were collected using a survey questionnaire in Chinese on the Wen Juan Xing^1^ platform. The study was conducted between 2023 and 2024, with a 6-months interval between waves. This design enabled temporal ordering of the focal variables, with T1 CSE and baseline learning burnout, T2 mediators, and T3 learning burnout, and provided longitudinal evidence relevant to early risk identification and mental health support for university freshmen.

For the present longitudinal analyses, inclusion criteria were: (1) enrollment as a first-year medical student at baseline; (2) baseline age between 18 and 25 years; (3) ability to complete the Chinese online questionnaire independently; and (4) provision of informed consent. Exclusion criteria were: (1) refusal to participate or withdrawal during the study; (2) failure to provide valid responses on key study variables; (3) failure to match data across the three waves; and (4) invalid responses identified during data quality screening (e.g., careless or duplicate responses).

This study was approved by the Ethics Committee for Scientific Research at the corresponding author’s affiliated institution (Approval No. 2023-021) and was conducted in accordance with the Declaration of Helsinki. Prior to participation, researchers provided participants with a comprehensive explanation of the study’s objectives, content, and procedures, ensuring that findings would be strictly utilized for academic research purposes. Participants were informed that all data collected would be anonymized and treated with strict confidentiality. Participants were informed that the data from this study would be anonymous and treated confidentially. They could stop participating at any time, and their participation was voluntary. Informed consent was obtained from participants before they completed the questionnaire, with participants signing informed consent forms.

### 2.2 Measures

#### 2.2.1 Socio-demographics

A demographic questionnaire elicited participant-related variables: age, gender (female or male), residence (rural or urban), and only-child status (only child or non-only child).

#### 2.2.2 CSE

Core self-evaluation was assessed using the Chinese version (32) of the Core Self-Evaluation Scale (12). The scale comprises 10 items with five-point answers (e.g., 1 = “complete disagreement,” 5 = “complete agreement”). Examples of the items include, “Sometimes I feel depressed” and “I complete tasks successfully.” The scale has good internal consistency (Cronbach’s alpha = 0.83), good test-retest reliability (*r* = 0.82), and criterion-related validity (32, 33). In this study the Cronbach’s alpha for the scale was 0.90.

#### 2.2.3 Anxiety-related distress-related distress

To provide a comprehensive assessment of participants’ anxiety-related distress, this study utilized three indicators focusing on different contexts: fear of missing out (social-cognitive facet), social anxiety (interpersonal facet), and state anxiety (general emotional-physiological facet).

Fear of missing out. Fear of missing out was measured using the Chinese version (34) of the Fear of Missing Out scale (35). Each of the 10 items of the scale is rated on a five-point scale ranging from “never” (value = 1) to “always” (value = 5). Higher scores indicate greater fear of missing out. Sample items include, “I get worried when I find out my friends are having fun without me” and “When I go on vacation, I continue to keep tabs on what my friends are doing.” This scale has shown good reliability (Cronbach’s alpha = 0.93) and construct validity in Chinese sample (36, 37). Cronbach’s alpha for the scale was 0.83 in our study.

Social anxiety. The Chinese version (38) of the six-item social anxiety subscale of the Self-Consciousness Scale was used to assess social anxiety (39). Participants are asked to respond on a five-point Likert scale (from 1 = “not at all” to 5 = “extremely”). Higher scores are indicative of a higher level of social anxiety. Examples of the items include, “I get embarrassed very easily” and “I don’t find it hard to talk to strangers.” Previous research has shown that the scale has a good internal consistency (Coefficient Alpha = 0.75) (38) and a good test-retest reliability (*r* = 0.77) (39). This scale has been found to discriminate effectively between high-social-anxiety and low-social-anxiety undergraduate students (40). In this study, the Cronbach’s alpha for the scale was 0.86.

State anxiety. The Chinese version of the anxiety subscale of the Depression Anxiety Stress Scale–21 (DASS-21) was used to assess state anxiety (41, 42). The scale has 7 items and each item is rated on a 4-point Likert scale ranging from 0 (“did not apply to me at all”) to 3 (“applied to me very much”). Examples of the items include, “I was aware of dryness of my mouth” and “I felt I was close to panic.” Higher scores indicate a greater level of state anxiety. The scale has good internal consistency (Cronbach’s alpha = 0.80) and moderate convergent validity (41, 42). In the present study, the Cronbach’s alpha for the scale was 0.82.

To evaluate the validity of the anxiety-related distress construct, a confirmatory factor analysis (CFA) was conducted. The single-factor model, with Fear of Missing Out, social anxiety, and state anxiety as indicators, demonstrated an acceptable fit to the data (χ^2^/df = 17.28, CFI = 0.94, TLI = 0.93, RMSEA = 0.058). These results support the use of a latent composite variable to represent anxiety-related distress.

#### 2.2.4 Problematic digital media use

To evaluate participants’ engagement in problematic digital media use, two specific domains were assessed: short-video use and problematic online gaming. These two indicators represent the most prevalent forms of digital consumption among university students.

Short-video use. The Chinese version (43) of the Short-video Use Intensity Scale was utilized to assess participants’ short-video use (44). Each of the scale’s six items is rated on a five-point scale (from 1 = “totally disagree” “to 5 = “totally agree”). Higher scores reflect greater short-video use. Sample items include, “Sometimes I have an impression that I live two lives: one private and another virtual” and “I fear that I might meet some of my virtual friends in real life.” Past studies have demonstrated the scale’s strong internal reliability (Coefficient Alpha = 0.81) and structure validity (43, 44). In the present study, Cronbach’s alpha for the subscale was 0.82.

Problematizing online gaming. Problematizing online gaming was evaluated by using the Chinese version of Problematizing Excessive Online Gaming Scale (45, 46). All 5 items were rated from 1 (“strongly disagree”) to 5 (“strongly agree”). Higher scores indicate greater problematizing excessive online gaming. Sample items include, “I sometimes lose sleep because of the time I spend playing online games” and “I sometimes skip meals or delay my eating because I am busy playing online games.” This scale had satisfactory internal reliability (Cronbach’s alpha = 0.72) and structure validity (45, 46). In the present study, Cronbach’s alpha for the subscale was 0.88.

To assess the validity of the problematic digital media use construct, a CFA was performed. The single-factor model, comprising problematic short-video use and online gaming as indicators, showed an adequate model fit (χ^2^/df = 53.47, CFI = 0.92, TLI = 0.90, RMSEA = 0.074). These findings support the use of a latent composite construct for problematic digital media use in subsequent analyses.

#### 2.2.5 Learning burnout

Learning burnout was assessed using the Chinese version of the Learning Burnout Scale (2). Each of these 20 items is rated on a five-point Likert scale (1 = “completely disagree” to 5 = “completely agree”). Examples of the items include, “Mastering professional knowledge is easy for me” and “I feel exhausted after studying all day.” Higher scores indicate greater learning burnout. Previous research shows that the scale has high internal consistency (Cronbach’s alpha = 0.93) and structure validity (2, 47). In the present study, the Cronbach’s alpha for the scale at T1/T3 was 0.91/0.91.

### 2.3 Data analysis

Descriptive statistics, Pearson correlation analyses, and attrition analyses were conducted using SPSS 26.0. Attrition analyses compared participants who completed all three waves with those who had missing data in at least one wave on key baseline demographic and study variables.

Structural equation modeling (SEM) with maximum likelihood estimation was performed in Mplus 8.3 to evaluate the revised baseline-adjusted parallel mediation model. In this model, T1 learning burnout was included as a baseline control variable, and T2 anxiety-related distress and T2 problematic digital media use were specified as parallel mediators linking T1 CSE to T3 learning burnout. The two T2 mediators were allowed to covary because they were measured at the same time point. A bootstrap procedure with 5,000 resamples was used to test the indirect effects. Indirect effects were considered statistically significant when the 95% confidence intervals did not include zero.

To assess common method bias, Harman’s single-factor test was first conducted. Given the limitations of this approach, an additional CFA-based test was performed by adding an unmeasured latent method factor to the measurement model.

A series of sensitivity analyses were conducted to examine the robustness of the findings. First, the mediation model was re-estimated while controlling for age, gender, residence, and only-child status. Second, multi-group analyses were conducted to examine whether the structural paths differed across gender, residence, and only-child status groups. For the gender multi-group analysis, measurement invariance was first examined before structural path comparisons. Third, the model was re-estimated using the full baseline sample (*N* = 5,614) with FIML. Model fit was assessed using the chi-square statistic (χ^2^), Comparative Fit Index (CFI), Tucker–Lewis Index (TLI), and Root Mean Square Error of Approximation (RMSEA). Values of CFI and TLI greater than 0.90 and RMSEA less than 0.08 were considered acceptable (48).

## 3 Results

### 3.1 Common method biases test

To assess the potential presence of common method bias (CMB), Harman’s single-factor test was first conducted. The unrotated factor analysis indicated that the first principal factor accounted for 22.75% of the total variance, which is below the commonly recommended threshold (49). Given the limitations of Harman’s single-factor test, we further conducted a more rigorous CFA-based assessment by adding an unmeasured latent method factor to the measurement model (50, 51). The model without the latent method factor showed the following fit indices: χ^2^/df = 13.95, CFI = 0.79, TLI = 0.78, RMSEA = 0.051. After including the latent method factor, the fit indices were χ^2^/df = 13.96, CFI = 0.79, TLI = 0.78, RMSEA = 0.051. The inclusion of the method factor did not result in a meaningful improvement in model fit, suggesting that CMB was unlikely to have substantially affected the main findings.

### 3.2 Attrition analysis

A total of 5,614 full-time first-year medical students were assessed at Time 1 (T1), 5,545 at Time 2 (T2), and 4,959 at Time 3 (T3). During the data collection period, 724 participants dropped out, resulting in 4,890 participants who completed all three waves. Attrition was primarily due to students being absent from school or transferring to another school at the time of data collection.

A formal attrition analysis was conducted by comparing participants who completed all three waves (*n* = 4,890) with those who had missing data at one or more waves (*n* = 724). The results showed that non-completers were older (*t* = −9.48, *p* < 0.001), more likely to be female (χ^2^ = 3.89, *p* = 0.049), and exhibited lower levels of social anxiety (*t* = 4.40, *p* < 0.001) and fear of missing out (*t* = 2.46, *p* = 0.014). No significant differences were found in residence (χ^2^ = 2.39, *p* = 0.122), only-child status (χ^2^ = 0.01, *p* = 0.965), CSE (*t* = −0.15, *p* = 0.884), state anxiety (*t* = 0.20, *p* = 0.843), short-video use (*t* = 0.92, *p* = 0.359), problematizing excessive online gaming (*t* = 0.05, *p* = 0.959), and learning burnout (T1/T3: *t* = 0.01/0.79, *p* = 0.996/0.432). Missing data were handled using Full Information Maximum Likelihood (FIML), which uses all available data to generate unbiased and efficient parameter estimates (52). The age of the final analytic sample ranged from 18 to 25 years, with a mean of 19.25 years and a standard deviation of 0.65 years. Detailed socio-demographic characteristics are presented in Table 1.

**TABLE 1:** Socio-demographic characteristics of the participants (*N* = 4,890).

Demographic information	*n*	Percentage (%)
Age, mean ± SD	–	19.25 ± 0.65
Gender		
Male	1,978	40.4
Female	2,912	59.6
Only child status		
Only child	1,821	37.2
Non-only child	3,069	62.8
Residence		
Rural	1,502	30.7
Urban	3,388	69.3

### 3.3 Descriptive statistics and correlations

Means, standard deviations and Pearson correlations between the study variables are presented in Table 2. As expected, T1 CSE was negatively correlated with T2 fear of missing out, T2 social anxiety, T2 state anxiety, T2 short-video use, T2 problematic online gaming, and both T1 and T3 learning burnout (*rs* = −0.71 to −0.24, *ps* < 0.001). T2 fear of missing out, T2 social anxiety, and T2 state anxiety were all positively correlated with T2 short-video use, T2 problematic online gaming, and both T1 and T3 learning burnout (*rs* = 0.18–0.37, *ps* < 0.001). In addition, T2 short-video use and T2 problematic online gaming were positively correlated with both T1 and T3 learning burnout (*rs* = 0.31–0.42, *ps* < 0.001).

**TABLE 2:** Descriptive statistics and correlations between variables (*N* = 4,890).

Variables	1	2	3	4	5	6	7	8	9	10	11	12
1. Age	1	1	1	1	1	1	1	1	1	1	1	1
2. Gender	−0.02											
3. Residence	−0.08***	−0.07***										
4. Only-child status	−0.03*	−0.14***	0.19***									
5. T1 CSE	0.01	−0.09***	0.07***	0.08***								
6. T2 FOMO	−0.04**	0.06***	0.02	−0.01	−0.24***							
7. T2 SA	−0.01	0.06***	−0.07***	0.08***	−0.42***	0.30***						
8. T2 SAN	−0.03*	0.02	−0.03	−0.04**	−0.37***	0.32***	0.34***					
9. T2 SVU	−0.03*	0.03*	−0.05***	−0.09***	−0.38**	0.32***	0.34***	0.35***				
10. T2 PEOG	0.01	−0.21***	−0.02	−0.01	−0.27***	0.19***	0.18***	0.29***	0.56***			
11. T1 LB	0.01	0.05***	−0.08***	−0.08***	−0.71***	0.21***	0.37***	0.30***	0.42***	0.32***		
12. T3 LB	−0.02	0.05***	−0.06***	−0.07***	−0.46***	0.22***	0.36***	0.31***	0.40***	0.31***	0.59***	
*M*	19.25				36.10	18.73	19.10	4.02	12.66	7.41	52.74	53.57
SD	0.65				6.91	5.92	5.02	5.32	4.69	3.31	11.09	11.74

Gender is a category variable (0 = male, 1 = female); Residence is a category variable (0 = rural, 1 = urban); Only child status is a category variable (0 = non-only child, 1 = only child); FOMO, fear of missing out; SA, social anxiety; SAN, state anxiety; SVU, short-video use; PEOG, problematizing excessive online gaming; CSE, core self-evaluation; LB, learning burnout; M, mean; SD, standard deviation,

*p < 0.05,

**p < 0.01,

***p < 0.001.

### 3.4 Testing for the mediation model

**FIGURE 2:**
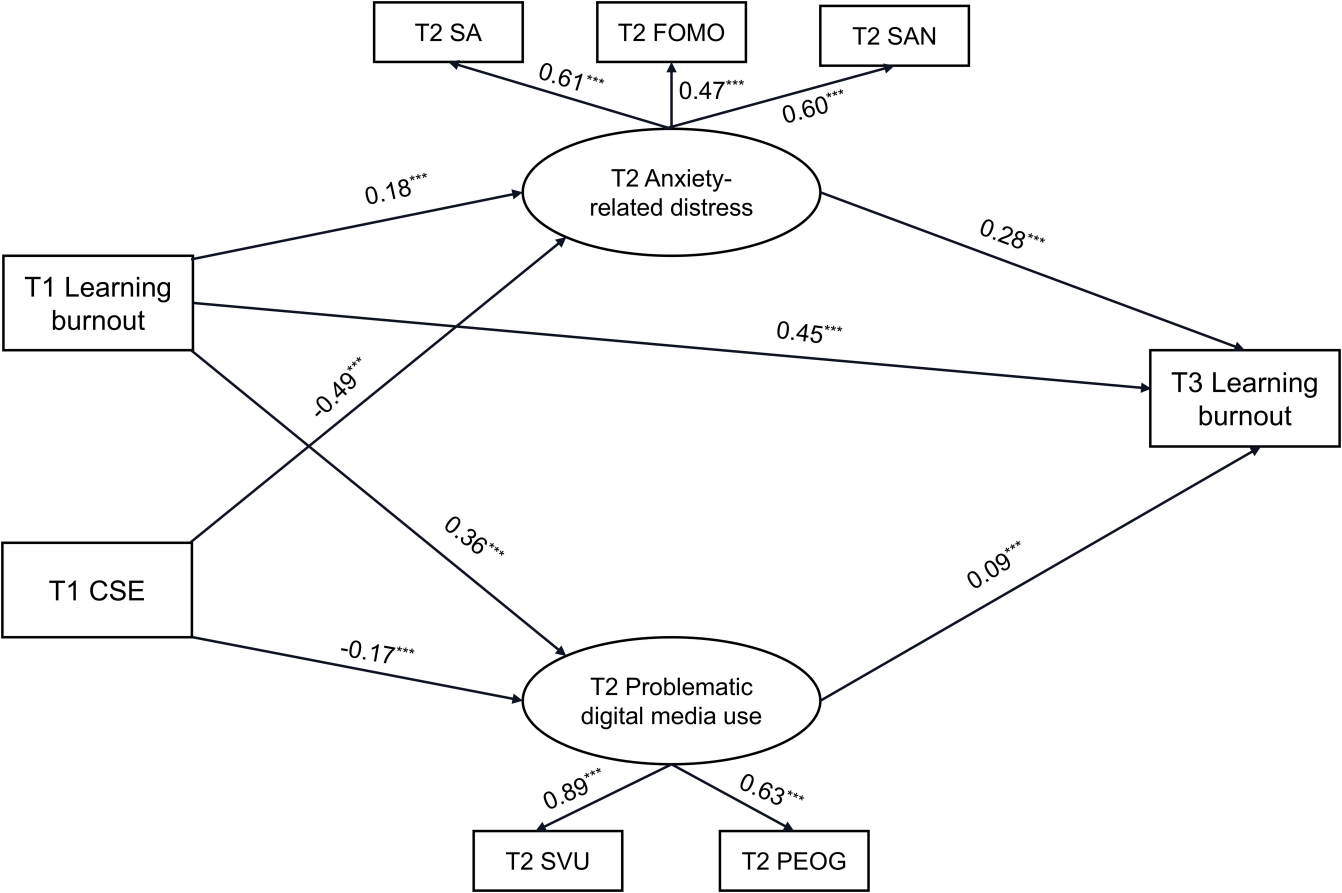
The standardized results of the baseline-adjusted parallel mediation model. For clarity, only significant standardized paths are shown; within-wave correlations and non-significant paths were estimated but are omitted from the figure. FOMO, fear of missing out; SA, social anxiety; SAN, state anxiety; SVU, short-video use; PEOG, problematic online gaming. ***p < 0.001.

Figure 2 presents the standardized results of the revised baseline-adjusted parallel mediation model. The model showed a good fit to the data (χ^2^/df = 16.49, CFI = 0.98, TLI = 0.95, RMSEA = 0.056). T1 learning burnout positively predicted T2 problematic digital media use (β = 0.36, *p* < 0.001) and T2 anxiety-related distress (β = 0.18, *p* < 0.001), and T3 learning burnout (β = 0.45, *p* < 0.001). T1 CSE negatively predicted T2 problematic digital media use (β = −0.17, *p* < 0.001) and T2 anxiety-related distress (β = −0.49, *p* < 0.001). Meanwhile, T2 anxiety-related distress (β = 0.28, *p* < 0.001) and T2 problematic digital media use (β = 0.09, *p* < 0.001) positively predicted T3 learning burnout. However, the direct path from T1 CSE to T3 learning burnout was not statistically significant.

Bootstrap analyses further indicated that the indirect effect of T1 CSE on T3 learning burnout through T2 anxiety-related distress was significant (effect = −0.14, 95% CI = [−0.18, −0.10]). The indirect effect through T2 problematic digital media use was also significant (effect = −0.02, 95 % CI [−0.03, −0.01]). These findings suggest that, after controlling for baseline learning burnout, the association between T1 CSE and later learning burnout was primarily reflected through indirect pathways via anxiety-related distress and problematic digital media use.

### 3.5 Sensitivity analyses

First, the parallel mediation model was re-estimated after controlling for covariates (i.e., age, gender, and only-child status), and the overall pattern of results remained unchanged. Second, to examine the robustness of the findings across subgroups, multi-group analyses were conducted. For the gender multi-group analysis, we first re-examined the measurement model across gender groups. Based on the modification indices and theoretical considerations–specifically, prior evidence indicating that males tend to report higher levels of problematic online gaming than females (53)–the intercept of problematic online gaming was freely estimated across gender groups. After this adjustment, the measurement model showed acceptable fit and supported configural and metric invariance, as well as partial scalar invariance, across gender (54). On this basis, structural path comparisons were conducted. The results indicated that constraining the structural paths to be equal across gender did not significantly worsen model fit, suggesting no substantial gender differences in the longitudinal associations among the study variables (see Table 3). Similar results were observed for residence status and only-child status groups. Third, we re-estimated the model using the full sample (*N* = 5,614) with FIML. The pattern and significance of the associations among the study variables remained largely unchanged, suggesting that attrition did not substantially bias the main findings. Taken together, these sensitivity analyses support the robustness of the revised results.

**TABLE 3:** The results of multi-group analysis across gender, residence, and only-child status groups.

Model	*χ ^2^*	*df*	CFI	TLI	RMSEA	*Δχ ^2^*	*Δ df*	*p*
Gender								
M1	329.42	31	0.97	0.94	0.063	4.97	5	0.420
M2	334.39	36	0.97	0.95	0.058			
Residence								
M1	271.20	32	0.97	0.95	0.055	4.68	5	0.456
M2	275.88	37	0.97	0.96	0.051			
Only-child status								
M1	265.44	32	0.97	0.96	0.055	4.58	5	0.469
M2	270.02	37	0.97	0.96	0.051			

M1 = Model without constraints on paths across gender, residence, and only-child status groups. M2 = Model with paths constrained to be invariant across gender, residence, and only-child status groups.

## 4 Discussion

This study used a three-wave longitudinal design to examine the associations among CSE, anxiety-related distress, problematic digital media use, and learning burnout in Chinese first-year medical students. After controlling for baseline learning burnout, the direct path from T1 CSE to T3 learning burnout was no longer significant. However, T1 CSE remained indirectly associated with T3 learning burnout through both T2 anxiety-related distress and T2 problematic digital media use. These findings suggest that, after accounting for initial burnout levels, the association between CSE and later learning burnout was primarily reflected through emotional and behavioral pathways rather than through a remaining direct effect.

Inconsistent with the first hypothesis, one important finding of the baseline-adjusted model was that, after T1 learning burnout was included as a baseline control variable, the direct path from T1 CSE to T3 learning burnout was no longer significant. This result suggests that baseline learning burnout accounted for a substantial proportion of variance in later learning burnout. Once this baseline component was taken into account, CSE no longer explained additional direct variance in T3 learning burnout. Theoretically, this does not imply that CSE is unimportant. Rather, it suggests that the influence of CSE may not be expressed through an independent direct pathway, but instead may operate through subsequent psychological and behavioral processes that shape later academic adaptation. This interpretation is not inconsistent with SDT. On the contrary, it suggests that CSE, as an important psychological resource, may exert its role primarily through indirect pathways within a more stringent longitudinal framework (55).

Aligned with Hypothesis 2, the present study further showed that anxiety-related distress served as a significant indirect pathway linking CSE to later learning burnout. This suggests that lower CSE may not influence subsequent learning burnout directly, but may first be reflected in higher levels of anxiety-related distress, which in turn are associated with later burnout. From the perspective of stress and coping theory (18), students with lower CSE may be more likely to appraise the high demands, rapid pace, and uncertainty of medical training as threats rather than challenges, thereby becoming more vulnerable to persistent tension, worry, and psychological strain. This vulnerability may be especially pronounced among first-year medical students, who are undergoing a rapid transition from general educational settings to professional medical training. In turn, anxiety-related distress may further deplete emotional and cognitive resources, weaken learning engagement, sustained attention, and adaptive self-regulation, and thereby increase the risk of later learning burnout. This finding is consistent with previous research linking psychological distress, anxiety, and academic burnout in medical and nursing students (6, 22), and is also compatible with evidence showing that academic anxiety is closely tied to academic burnout in university students (23). These results suggest that early support for first-year medical students should attend not only to burnout itself but also to anxiety-related distress at an earlier stage.

The study also found support for the hypothesis 3, problematic digital media use served as a significant indirect pathway linking CSE to later learning burnout. This suggests that lower CSE may be associated not only with anxiety-related distress but also with later learning burnout through problematic digital engagement as a behavioral pathway. For students with lower CSE, reduced feelings of competence and self-worth may increase the tendency to rely on highly immersive and immediately rewarding digital activities, such as short-video use and online gaming, for temporary distraction, compensation, or emotional relief. When such digital media use becomes characterized by impaired control, escapist motivation, and excessive immersion, it may further consume study time, weaken self-regulatory capacity, disrupt sleep, and interfere with sustained attention, thereby increasing the risk of later learning burnout. This finding is broadly consistent with previous research linking problematic internet use, problematic smartphone use, and academic maladjustment or burnout (56, 57), and is also compatible with SDT (14), which suggests that reduced psychological resources may promote compensatory behaviors. These results further imply that support for first-year medical students should address not only anxiety-related distress but also maladaptive patterns of digital media engagement under conditions of academic stress.

Despite the use of a three-wave longitudinal design and the inclusion of baseline learning burnout in the model, several limitations should be noted. First, participants were recruited exclusively from four medical universities in Zhejiang Province, China, which may limit the generalizability of the findings to other regions, medical education contexts, or broader health science student populations. Second, the main variables were assessed through self-report measures. Although we supplemented the original analysis with a more rigorous assessment of common method bias, response bias associated with self-report data cannot be completely ruled out. Third, although anxiety-related distress and problematic digital media use were modeled as parallel pathways in the present study, they were still assessed at the same time point, and the present design therefore cannot clarify their dynamic relation or potential temporal ordering. Fourth, both anxiety-related distress and problematic digital media use were treated as relatively broad latent domains; future research may benefit from examining whether their specific components operate differently. Finally, although the present study used a longitudinal design, controlled for baseline learning burnout, and handled missing data using FIML, it remains observational in nature and therefore does not support strict causal inference. Future studies using additional waves, more intensive longitudinal designs, and multi-method assessments may help further clarify the dynamic relations among CSE, anxiety-related distress, problematic digital media use, and learning burnout.

## 5 Implications

The present study has both theoretical and practical implications. Theoretically, after controlling for baseline learning burnout, the association between CSE and later learning burnout was primarily reflected through two indirect pathways: anxiety-related distress and problematic digital media use. This pattern suggests that, among first-year medical students, the role of CSE may be better understood in terms of its influence on subsequent emotional adaptation and behavioral regulation rather than as a direct effect on learning burnout itself. In this sense, the present findings provide a more process-oriented perspective on medical student burnout, indicating that personality-related resources, emotional distress, and maladaptive digital engagement may jointly contribute to later academic maladjustment.

Practically, these findings suggest that universities, particularly medical schools, should not focus only on the overt manifestations of learning burnout, but should also identify students at earlier stages who show lower CSE, elevated anxiety-related distress, or problematic digital media use. For such students, more targeted support may be warranted during the transition into university life, including interventions designed to strengthen competence and self-efficacy, training in anxiety management and emotional regulation, and guidance on the use of highly immersive digital media such as short-video platforms and online gaming. By addressing both emotional and behavioral risks, institutions may be better positioned to reduce the likelihood of later learning burnout and support a smoother transition from general education to professional medical training.

## 6 Conclusion

Using a three-wave longitudinal design, the present study examined the associations among core self-evaluation, anxiety-related distress, problematic digital media use, and later learning burnout in first-year medical students after accounting for baseline learning burnout. The results showed that the direct path from CSE to later learning burnout was no longer significant, whereas significant indirect pathways were observed through anxiety-related distress and problematic digital media use. These findings suggest that the association between CSE and later learning burnout is primarily reflected through emotional and behavioral mechanisms. The present study provides more rigorous longitudinal evidence on early psychological risk factors underlying learning burnout in first-year medical students and offers useful implications for earlier and more targeted identification and intervention in medical education.

## Data Availability

The raw data supporting the conclusions of this article will be made available by the authors, without undue reservation.

## Footnotes

1
www.wjx.cn
